# Chitosan-Polylactide/Hyaluronic Acid Complex Microspheres as Carriers for Controlled Release of Bioactive Transforming Growth Factor-β1

**DOI:** 10.3390/pharmaceutics10040239

**Published:** 2018-11-17

**Authors:** Qing Min, Jiaoyan Liu, Jing Li, Ying Wan, Jiliang Wu

**Affiliations:** 1 School of pharmacy, Hubei University of Science and Technology, Xianning 437100, China; baimin0628@163.com; 2 College of Life Science and Technology, Huazhong University of Science and Technology, Wuhan 430074, China; liujiaoyan@hust.edu.cn; 3 Hubei Province Key Laboratory on Cardiovascular, Cerebrovascular and Metabolic Disorders, Hubei University of Science and Technology, Xianning 437100, China; lijing790629@163.com

**Keywords:** chitosan, polylactide, hyaluronic acid, microspheres, delivery of bioactive molecules, transforming growth factor-β1

## Abstract

Chitosan(CH)-polylactide(PLA) copolymers containing varied PLA percentages were synthesized using a group-protection method and one of them with solubility in water-based solvents was used to prepare CH-PLA/hyaluronic acid (HA) complex microspheres for the delivery of transforming growth factor-β1 (TGF-β1). An emulsification processing method was developed for producing TGF-β1-loaded CH-PLA/HA microspheres using sodium tripolyphosphate (TPP) as ionic crosslinker and the size of the microspheres was devised to the micron level in order to achieve high encapsulating efficiency. The encapsulating efficiency, swelling property and release administration of the microspheres could be synergistically regulated by PLA component, the applied TPP dose and the incorporated HA amount. In comparison to CH/HA microspheres, the CH-PLA/HA microspheres had greatly reduced TGF-β1 release rates and were able to administrate the TGF-β1 release at controlled rates over a significant longer period of time. The released TGF-β1 was detected to be bioactive when compared to the free TGF-β1. These results suggest that the presently developed CH-PLA/HA complex microspheres have promising potential in delivering TGF-β1 for cartilage repair applications where the applied TGF-β1 amount in the early stage needs to be low whilst the sustained TGF-β1 release at an appropriate dose in the later stage has to be maintained

## 1. Introduction

Cartilaginous tissues play important roles in contributing certain crucial functions such as very low wear resistance, load bearing and energy dissipation in joints of the musculoskeletal system. These tissues have a limited capacity for self-repair after injury owing to its avascular and alymphatic nature as well as low cell density [1]. Currently used clinical therapies for cartilage repair mainly include microfracture, subchondral bone drilling and mosaicplasty and autologous or allologous implantation. However, these techniques have their respective shortcomings and in particular, many cases involving the mentioned surgical interventions have not proven to be successful from a long-term point of view [2,3].

Tissue-engineering strategies have become attractive for their potential to repair cartilage lesions by using degradable scaffolds, exogenous bioactive molecules, and/or cells [2,4]. To date, a wide variety of scaffolds in the form of sponges, films, fibers and hydrogels have been developed [5]. In addition to the important role of the scaffold, many studies have suggested that the local presence of appropriate bioactive molecules at the requisite concentrations is of particular importance to the cases of cartilage repair since these molecules are able to induce the recruitment and proliferation of the cells that migrate from the synovial membrane and subsynovial space and to transform the recruited cells into chondrocytes [6,7,8]. Among the bioactive molecules applicable for cartilage repair, transforming growth factor-β1 (TGF-β1) can act in both capacities with mitosis and chemotaxis, being regulated by the factor concentration and the factor action time period [7,8]. In general, ectogenic TGF-β1 is not systematically administered in vivo because it is short-lived when exposed to a physiological environment. Proper carriers are usually required to protect TGF-β1 from proteolysis or antibody neutralization and to locally deliver it at effective concentrations and controlled rates [1,6,8,9].

Synthetic polyesters with biodegradable nature were commonly employed as vehicle materials to build carriers for delivery of protein growth factors [10]. Nevertheless, their high hydrophobicity, lack of functional groups and slow degradation rate limit their capability for the factor release administration [7,10,11]. In addition, polyester-based vehicles are generally constructed by means of organic solvents, which could possibly attenuate the bioactivity of the loaded factors [7,10]. Natural polymers, including collagen, gelatin, fibrin, silk fibroin, hyaluronic acid, alginate and chitosan (CH), have been investigated as alternative carrier materials for the delivery of growth factors [6,7,10]. Among these mentioned natural polymers, CH is a widely used one as it has many unique properties such as biodegradability, hydrophilicity, antimicrobial activity, bioadherence and cell affinity [12,13,14]. CH microspheres crosslinked by certain ionic crosslinkers (e.g., sulfate, citrate and sodium tripolyphosphate) have been used as carriers for delivery of TGF-β1 [10,13,15]. Since the mentioned anionic crosslinkers are nontoxic and the linkages among CH molecular chains are formed mainly basing on electrostatic interactions, the bioactivity of TGF-β1 released from CH microspheres can thus be well preserved [10,13]. Nevertheless, such constructed CH microspheres showed severe initial burst release features and had a limited ability to control the release of TGF-β1 at a proper level over a long enough period of time [10].

In recent years, polyelectrolyte composites have emerged as effective delivery systems and many new processing techniques have been developed for constructing polyelectrolyte composite carriers [16]. Polyelectrolyte complexes composed of positively charged CH and negatively charged natural polymers such as alginate, hyaluronic acid (HA) and pectin have aroused considerable interest in the field of drug delivery [17]. Such types of polyelectrolyte complexes are usually non-toxic and biocompatible because the interactions between polyanionic molecules and polycationic molecules majorly involve electrostatic association and/or hydrogen and hydrophobic bonds. Among the enumerated polyanionic polymers, HA is often used together with CH for constructing CH/HA polyelectrolyte complexes [17]. HA is a liner glycosaminoglycan composed of N-acetyl-D glucosamine and d-glucuronic acid units and it exists in many types of extracellular matrixes (ECMs) in human body as an important component for facilitating cell locomotion, proliferation and phenotype preservation [18,19]. HA shows excellent potential for application in drug delivery, wound healing and tissue engineering due to its good biocompatibility, biodegradability and mucoadhesion [15,17,18,19]. CH/HA complexes were also used as gene vectors and the optimal complex showed significantly higher transfection efficiency for different targeting cells as compared to the vectors comprised of CH only [17,20,21,22].

Several studies on CH/HA complex nanoparticles point to their utilization in delivery systems [17]. In this study, an attempt was made to develop a new type of CH-based complex microspheres for TGF-β1 delivery by using amphiphilic chitosan-polylactide (CH-PLA) copolymers and HA. CH-PLAs were synthesized by grafting PLA side chains onto the C-6 sites of CH backbone while leaving the amino groups at the C-2 sites free using a group-protection method. The optimal CH-PLA with solubility in waster-based solvents was combined with HA to construct TGF-β1-loaded CH-PLA/HA complex microspheres in the presence of sodium tripolyphosphate (TPP) as crosslinker. Since CH-PLAs contain hydrophobic PLA side chains but their amino groups are free, CH-PLA/HA complexes could still be formed via the interactions between the amino groups in CH backbone and the carboxyl groups in HA. In addition, an emulsification preparation method was developed for producing TGF-β1-loaded CH-PLA/HA microspheres with their size at micron level in order to achieve high encapsulating efficiency. So produced CH-PLA/HA microspheres were found to show significantly improved capacity for reducing the initial fast release of TGF-β1 and for prolonging the TGF-β1 release at controlled rates in comparison to CH/HA microspheres, making them suitable for the use in cartilage repair [16,17,18]. Some results related to the preparation and characterization of the microspheres and to the TGF-β1 release patterns as well as its bioactivity preservation were reported.

## 2. Materials and Methods

### 2.1. Materials

CH powder was procured from Aladdin Inc (Shanghai, China). Deacetylation degree and viscosity average molecular weight of CH were measured as 94.2 (±1.7)% and 1.12 (±0.14) × 10^5^, according to the reported methods [23]. Human recombinant TGF-β1 and TGF-β1 Quantikine ELISA Kit were supplied by PeproTech Inc (Rocky Hill, NJ, USA) and R&D Systems (Minneapolis, MN, USA), respectively. HA (sodium salt, M_w_:90–110 kDa), lactide (LA) and polyvinyl alcohol (PVA, 87–89% hydrolyzed, M_w_:13–23 kDa) were purchased from Sigma-Aldrich (Shanghai, China). XTT tetrazolium assay kit was purchased from Trevigen (Gaithersburg, MD, USA). All other reagents and chemicals were of analytical grade and purchased from Sinopharm, Shanghai, China.

### 2.2. Synthesis of CH-PLA Copolymers

CH-PLA copolymers were synthesized using a group-protection method [24,25]. In brief, the sifted CH powder (106–150 µm; 1.5 g) and phthalic anhydride (4.1 g) were dispersed in 30 mL of dried DMF and the mixture was heated at 90 °C for 8 h with stirring to produce phthaloyl-chitosan (PHCH). After reaction, the product was washed with anhydrous ethanol and dried to constant weight. In a typical procedure for synthesizing CH-PLA, LA (2.23 g) and PHCH (1.0 g) were added to a flask dried at reduced pressure for 1 h prior to the addition of toluene (10 mL). The reaction was carried out at 100 °C in nitrogen atmosphere for varied durations up to 60 h. After that, the flask was cooled down to ambient temperature and the obtained mixture was washed with 20 mL of acetone to remove the unreacted LA monomer and the PLA homopolymer. The collected product was then extracted with acetone for at least 12 h to obtain phthaloyl-chitosan-polylactide (PHCH-PLA). The obtained PHCH-PLA was deprotected using hydrazine monohydrate to produce chitosan-polylactide (CH-PLA). By mainly changing the feed ratio of LA to PHCH, CH-PLAs with various PLA percentages were synthesized. To ensure potential solubility of CH-PLAs in aqueous media, CH-PLA with a PLA content of about 38 wt % was used to prepare CH-PLA/HA complex microspheres.

### 2.3. Preparation of Microspheres

An emulsification method was used to prepare microspheres using TPP as a crosslinker. The preparation method is briefly described as follows. The selected CH-PLA was dissolved in 1% acetic acid aqueous solution to prepare 1 wt % CH-PLA solutions. To each solution, a given amount of TGF-β1 in 4 mM HCl was introduced, followed by adding PVA to reach a PVA concentration of 0.5 wt %. HA aqueous solutions with different concentrations (0.15 and 0.3 wt %) were also prepared. Each HA solution was added with PVA and TPP so that the concentration of PVA was 0.5 wt % and the ratio of TPP to the matrix of intended complex microspheres was 2 and 4, respectively. After that, a CH-PLA composite solution and one of HA composite solutions were isometrically introduced to a vessel containing mineral oil to reach a volume ratio of 7 (oil phase):1(aqueous phase). The mixture was homogenized at 8000 rpm for 30 min. The resulting emulsion was centrifuged at 7000 rpm for 10 min to separate the aqueous phase and the oil phase and CH-PLA/HA microspheres were collected by further centrifugation. The microspheres were washed with petroleum ether, alcohol and water in order, followed by lyophilization. CH microspheres and CH/HA microspheres were also produced with the same method and they were fed with the same amount of TGF-β1 during their preparation. These CH and CH/HA microspheres were used as controls.

### 2.4. Characterization

^1^H nuclear magnetic resonance (NMR) spectra were recorded on a spectrometer (Bruker AV 500, Rheinstetten, Germany) using a mixed solvent of D_2_O and CF_3_COOD (95:5, v/v). Elemental analyses were performed to determine the PLA content in the CH-PLAs using an elemental analyzer (Vario EL III, Elementar, Hanau, Germany). Microspheres were sputter-coated with gold and their morphology was viewed with a scanning electron microscope (SEM, Quanta 200, FEI, Eindhoven, the Netherlands). Fourier transform infrared (FTIR) spectra of CH-PLA samples were recorded on a spectrometer (VERTEX 70, Bruker, Ettlingen, Germany). For each kind of microspheres, their size-distribution was determined by measuring the diameters of 200 microspheres in their SEM images in a random manner and their average size was thus calculated.

### 2.5. Determination of TGF-β1 Encapsulating Efficiency

Microspheres were extracted using a 4 mM HCl solution with shaking (60 rpm) at 37 °C for 24 h. The collected supernatants were assayed to determine the TGF-β1 content in the microspheres by using a TGF-β1 Quantikine ELISA Kit and following the instructions provided by the manufacture. Encapsulating efficiency (*EE*) was calculated using following formula:

*EE* (%) = (*M_m_*/*M*_f_) × 100% (1)

where *M_m_* is the measured amount of TGF-β1 in the microspheres and *M*_f_, the feed amount of TGF-β1 during microsphere preparation.

### 2.6. Swelling Index

Weighed dry microspheres (*W_d_*) were immersed in a phosphate buffer saline (PBS) solution at 37 °C for 5 h. After that, they were transferred into some glass tubes having a sintered glass bottom and excess water was removed by centrifugation at 2000 rpm for 1 min. Weight (*W_s_*) of swollen microspheres was measured and their swelling index (*SI*) was calculated as follows:

*SI* (%) = [(*W_s_* − *W_d_*)/*W_d_*] × 100% (2)

### 2.7. In Vitro Release of TGF-β1

Release studies were conducted in PBS (pH 7.4). In brief, 10 mg of microspheres was introduced into a microcentrifuge tube filled with 0.25 mL of PBS (pH 7.4) and the tube was maintained at 37 °C. At predetermined time points, the tube was centrifuged to collect supernatant, replenished with fresh PBS and followed by vortex for 1 min. The released TGF-β1 amount in the supernatant was measured using the above mentioned TGF-β1 essay kit. All microsphere samples were tested with the same method and the average values reported were based on three specimens for each sample.

### 2.8. Activity Assessment of Released TGF-β1

Activity of TGF-β1 released from four kinds of microspheres (CH/HA-I(2), CH/HA-II(b) CH-PLA/HA-I(2) and CH-PLA/HA-II(b)) was assessed using the Mink lung cell growth inhibition assay [26]. Briefly, 10 mg of microspheres was placed in dulbecco’s modified eagle medium (DMEM), 250 µL) and kept at 37 °C. At each time point (day 1, 3, 7, 10, 14, 17 and 21), the supernatant was collected by centrifugation. The microsphere sample was refreshed with the same volume of DMEM and vortexed for 1 min. Supernatants matching with four kinds of microspheres were collected using the same method. Mink lung cells in DMEM were plated in 96-well culture dishes in triplicate at a density of 5 × 10^3^ cells/well, supplementing with 10% fetal calf serum. After attachment, the supernatants collected from different microsphere groups were added to the wells, respectively, and cells were further cultured for 48 h. The control wells were added with the same amount of free TGF-β1. Cell viability was assessed with the colorimetric XTT tetrazolium assay following the manufacturer’s instructions and using a standard curve with TGF-β1 concentrations in the range of 0.01–1 ng/mL. The level of cell growth inhibition was expressed as percentages related to the cells without TGF-β1 treatment. In addition, a certain amount of free TGF-β1 was stored in DMEM and kept at 37 °C for various periods up to 21 days. It was used as control for making activity comparisons between such treated free TGF-β1 and the released TGF-β1. 

### 2.9. Statistical Analysis

Data were presented as mean ± standard deviation. One-way analysis of variance was conducted and the level of statistical significance is defined as *p* < 0.05.

## 3. Results and Discussions

### 3.1. CH-PLA Characterization

**Figure 1:**
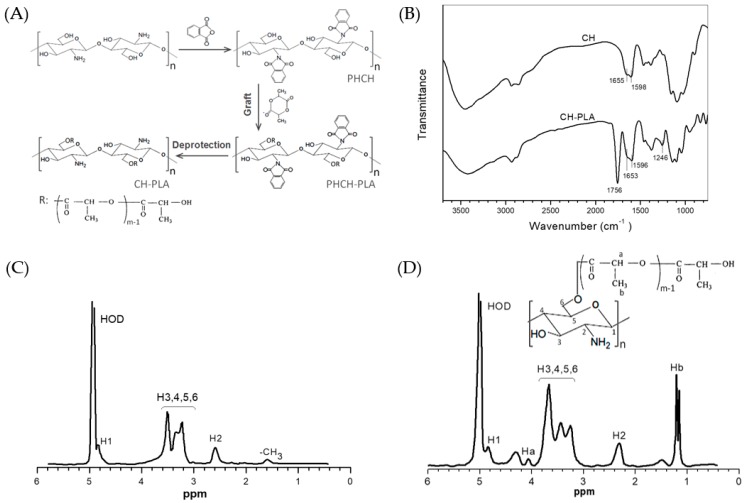
Schematic illustration (**A**) for synthesis of CH-PLA; FTIR spectra (**B**) of CH and CH-PLA; and ^1^H NMR spectra of CH (**C**) and CH-PLA (**D**) (PLA content in CH-PLA: 40.7 wt %).

In this study, PLA side chains were selectively grafted onto the C-6 sites of the CH backbone rather than the C-2 sites using phthalic anhydride as a group protective reagent in order to leave amino groups at the C-2 site free for the subsequent complex microsphere preparation and TPP crosslinking. A schematic illustration for synthesis of CH-PLA is presented in Figure 1A.

Figure 1B presents two FTIR spectra for CH and CH-PLA. A shoulder-like band at round 1655 cm^−1^ and another band at 1598 cm^−1^ are characteristic bands for CH with a relatively high deacetylation degree and they correspond to primary amine (N–H bending) and secondary amine (C=O stretching) of CH, respectively [27]. In the case of the spectrum for CH-PLA, two new bands are recorded at 1756 and 1249 cm^−1^ and they could be assigned to the carboxylic ester in PLA side chains [25,28]. Two other bands (1653 and 1596 cm^−1^) that originally belong to the amide I and amide II of CH are clearly registered for CH-PLA, suggesting that PLA side chains have been conjugated to the C-6 sites of CH chain units instead of their C-2 sites.

Figure 1C, D show ^1^H NMR spectra of CH and CH-PLA, respectively. Shifts recorded at about 2.59 (H-2), 3.2–3.5 (H-3, -4, -5, -6) and 4.8 (H-1) ppm are typically attributed to CH [25]. With respect to CH-PLA, a new signal appeared at ca. 1.2 ppm can be ascribed to the methyl protons located at the terminal groups and the backbone of the polylactide moiety. Another new singlet signal at about 4.1 ppm belongs to the protons in the repeat units of PLA chains [24,25]. CH-PLA has the characteristic signals for the CH component with small shifts in its spectrum and the signal area ratio of H-2 to H-1 is detected to be nearly the same as that for CH, corroborating that the PLA side chains are grafted onto the C-6 sites of CH backbone. On the basis of presented FTIR and ^1^H NMR spectra, it can be reached that so synthesized CH-PLA possesses the designed structures.

Several kinds of CH-PLAs with various PLA percentages were synthesized by mainly changing the ratio of LA to PHCH, reaction time and the volume of reaction media while keeping other reaction conditions constant and their relevant parameters are listed in Table 1. It is known that PLA is hydrophobic polyester with notably slower degradation rate as compared to CH [24,29]. When certain CH-PLAs with soluble nature in aqueous media are used to produce CH-PLA/HA complex microspheres for delivery of drugs or active molecules, the PLA side chains in the CH-PLAs would facilitate to drag the loaded drugs or molecules and thereby, to reduce their release rates, implying that the higher PLA content in the CH-PLAs will be potentially advantageous for improving the release administration of CH-PLA/HA microspheres. Based on a number of preparatory trials, it was found that CH-PLAs with a PLA percentage higher than 80 wt % could be achieved under the present synthesis conditions. Nevertheless, data in Table 1 exhibit that the CH-PLA with a PLA content of around 46 wt % is insoluble in a 1% acetic acid aqueous solution, which means that this kind of CH-PLA is unsuitable for the subsequent preparation of CH-PLA/HA complex microspheres. In order to ensure the full solubility of CH-PLAs in aqueous media while taking advantage of PLA component in CH-PLAs, CH-PLA (2) was selected for the following microsphere preparation.

### 3.2. Parameters of Microspheres

CH is a cationic polymer and it is able to form ionic complexes with certain anionic substances [16]. In the light of anionic features of HA, CH/HA nanoparticles can be produced in aqueous media via blending method while altering the component proportions and the concentrations of component solutions [15,16,17,30]. Despite the various applications of CH-based complex nanoparticles, they generally have low *EE* when used for delivering hydrophilic drugs or biomolecules [14,15,30]. Considering that the presently devised microspheres will be used for cartilage repair by injection and their *EE* needs to be high for saving expensive TGF-β1, their size can thus be designed to the micron level instead of the nano level. In view of structure and property of CH-PLA copolymers, the technique used for the preparation of CH/HA nanoparticles seems to be applicable for the preparation of CH-PLA/HA complex microspheres since CH-PLA has the similarity to CH in the presence of free amino groups at the C-2 sites of CH backbone. Disappointedly, it was found that simply blending a CH-PLA solution with a HA solution was unworkable for preparing CH-PLA/HA microspheres that have controlled morphologies and sizes even with the aid of different crosslinkers because CH-PLA and HA would fast form into quite irregular agglomerates when being blended in aqueous media. An emulsification approach was therefore developed for the preparation of CH-PLA/HA microsphere by using PVA as an emulsifier.

In general, multiple factors can influence the size, morphology, structure and property of the resulting microspheres when an emulsification technique is employed [16,31]. In the present instance, a number of preliminary experiments were conducted to optimize several major processing parameters, including concentration of solutions, stirring speed, the amount of the applied emulsifier and the volume ratio of dispersed phase and continuous phase. Therefrom, two sets of TGF-β1-loaded microspheres were prepared and relevant parameters for them are summarized in Table 2.

Up to now, varied kinds of covalent crosslinkers, such as glutaraldehyde, diisocyanate, genipin, ethylene glycol diglycidyl ether and glycidoxypropyltrimethoxy silane, have been utilized to crosslink CH [32,33,34]. Taking into consideration the requirements for the activity preservation of the loaded TGF-β1 in the resulting microspheres, in this study, TPP, an ionic crosslinker, was selected for crosslinking CH-PLA/HA microspheres because it can crosslink the protonated CH in acidic aqueous media via ionic interactions and also, is a safe and biocompatible reagent for bioactive molecules [31,32]. HA is a water-soluble polysaccharide and the HA content in the CH-PLA/HA microspheres was found to significantly affect the properties of the microspheres. A high HA content in CH-PLA/HA complexes could result in preponderant formation of irregular agglomerates rather than microspheres even though a liberal amount of TPP was applied. On the other hand, a high HA content in CH-PLA/HA microspheres would also cause the microspheres to markedly swell in aqueous media even if such microspheres could be achieved, which is quite disadvantageous for the microspheres to administer the release of the loaded TGF-β1. Therefore, the compositional proportions of CH-PLA/HA microspheres and the applied amount of TPP were optimized using the orthogonal testing method. The weight ratio of HA to CH-PLA/HA was thereby controlled at 0.3/1.0 or lower whereas the ratio of TPP to matrix was formulated as 2/1 and 4/1, respectively, as illustrated in Table 2.

**Figure 2:**
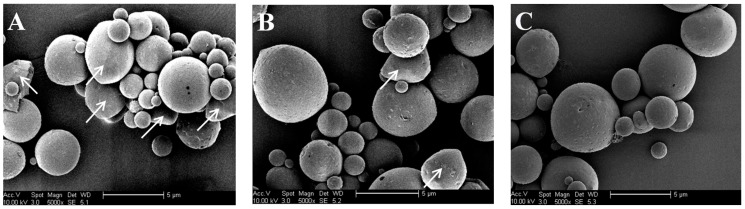
Representative scanning electron microscopy (SEM) micrographs for (**A**) CH-I; (**B**) CH/HA-II(b) and (**C**) CH-PLA/HA-II(b) microspheres (arrows denote the cracked clumps and irregularly shaped beads).

Representative SEM micrographs for different microspheres are presented in Figure 2. The image in Figure 2A shows that some CH-I microspheres with regular sphericity could be produced but some cracked clumps and irregularly shaped beads were also seen, as denoted by white arrows. Figure 2B displays that the fewer beads with irregular shapes were viewed as compared that shown in Figure 2A and many CH/HA-II(b) microspheres were spherical with smooth surface. The image for CH-PLA/HA-II(b) microspheres exhibits that most of them possessed regular sphericity. These images reveal that CH-PLA/HA microspheres with well-controlled morphology can be obtained under the present processing conditions while following the formulated compositions provided in Table 2.

**Figure 3:**
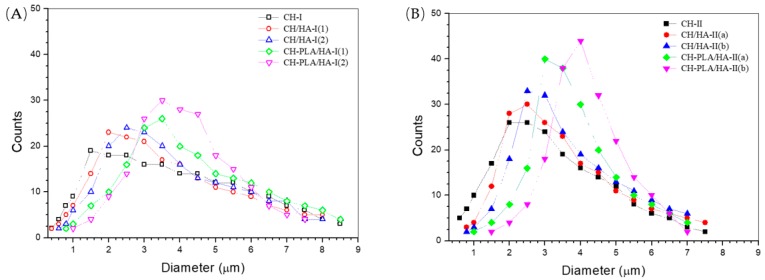
Variations in size-distribution of microspheres in set-one (**A**) and in set-two (**B**) (see Table 2 for their compositions).

Figure 3 depicts size-distributions for different kinds of microspheres. The microspheres in set-one had broad and scraggly size-distributions whilst the size-distributions for the microspheres in set-two became significantly narrowed with peaked characteristics. As shown in Table 2, the microspheres in set-one were crosslinked by TPP at a TPP/matrix ratio of 2/1, the applied TPP amount might not be enough to sufficiently crosslink these microspheres, which is indirectly confirmed by presence of cracked clumps and irregularly shaped beads (see Figure 2A), leading to their broad and fluctuating size-distributions. On the other hand, the microspheres in set-two were crosslinked with the redoubled amount of TPP as compared to that in set-one and these microspheres could thus be crosslinked to a relatively high degree. Accordingly, the microspheres in set-two would become denser than those crosslinked by a lower amount of TPP, resulting in their relatively narrowed size-distributions.

The effects of HA and PLA components on the size-distributions of microspheres can also be seen from Figure 3. Figure 3A exhibits that in contrast to CH-I microspheres, the size-distributions of CH/HA-I(1) and CH/HA-I(2) microspheres remained to be scraggly and asymmetric but the size-distributions for CH-PLA/HA-I(1) and CH-PLA/HA-I(2) microspheres showed somewhat symmetry. In Figure 3B, improved smoothness and better symmetry of size-distributions for CH/HA-II(a), CH/HA-II(b), CH-PLA/HA-II(a) and CH-PLA/HA-II(b) were clearly observed as compared to that for CH-II microspheres. These results indicate that (1) at a low TPP dosage (Figure 3A), the effect of HA component on the smoothness and shape symmetry of size-distributions is not significant but HA component together with PLA component could possibly improve the size-distribution symmetry of microspheres; and (2) at a high TPP dosage (Figure 3B), HA component alone or in conjunction with PLA component can help to smooth the size-distributions of microspheres and to reform their symmetry. It is known that symmetrical size-distribution and narrow distribution interval are two extremely needed characteristics for the desired polymer microsphere carriers since these two parameters are strongly correlated to the repeatable controlled release behavior of the microspheres [10,15,16]. Results in Figure 3 suggest that CH-PLA/HA-II(a) and CH-PLA/HA-II(b) microspheres should be optimal ones in terms of their size-distribution.

### 3.3. Encapsulating Efficiency and Swelling Property of Microspheres

Data numerated in Table 2 show that CH/HA microspheres had higher *EE* in comparison to their respectively matched CH microspheres, meaning that presence of HA component in microspheres or utilization of an increasing amount of TPP could help to increase *EE* of microspheres. Table 2 also elucidates that further rising *EE* can be achieved, given that the HA amount in the CH-PLA/HA microspheres is proper while the applied TPP is sufficient for crosslinking them, which is supported by *EE* of CH-PLA/HA-II(a) and CH-PLA/HA-II(b) microspheres. As described in the experimental section, TGF-β1 was loaded into CH or CH/HA microspheres via physical blending and the chain network formed inside microspheres was loose in the wet sate because these chains were held by TPP-associated ionic linkages and HA-involved electrostatic force. As a result, some amounts of TGF-β1 on the superficial layer of the CH or CH/HA microspheres would be easily washed away during the microsphere preparation, leading to their relatively low *EE*.

TGF-β1 is a type of protein factor with isoelectric point of around 8.6 [35] and it will be in positively charged state under neutral or acidic pH conditions. TGF-β1 molecular chains can thus easily interact with negatively charged HA chains during the microsphere preparation. On the other hand, negatively charged HA chains will also interact or entangle with CH-PLA chains because CH main chains contain a large amount of free amino groups. The short PLA side chains in CH-PLA are hydrophobic and they could possibly shape into heliciform hooks while being protruded from CH main chains when CH-PLA is exposed to aqueous media. As a result, PLA side chains will snag the TGF-β1, HA and CH chains to form entanglemants, which will certainly prevent the loss of TGF-β1 during the microsphere preparation and result in high *EE* for the resulting microspheres.

*SI* of hydrophilic polymer carriers is closely correlated to their capability for administrating the release of loaded drugs [36]. Hence, microspheres were measured to determine their *SI* and relevant data are listed in Table 2. It can be seen that (1) CH microspheres in both sets showed significantly larger *SI* than other kinds of microspheres and CH/HA microspheres in each set had slightly reduced *SI* when compared to CH microspheres; and (2) *SI* of CH-PLA/HA microspheres in each set was much smaller than that for CH or CH/HA microspheres. The large *SI* for CH microspheres is attributed to their high hydrophilicity and the loose chain network constructed by ionic bonding. CH contains many polar groups such as amino and hydroxyl groups and thus, intermolecular and intramolecular hydrogen bonds can easily form among CH chains in the dry state. However, these hydrogen bonds could be greatly weakened or even broken in the wet state [32] and consequently, hydrated CH microspheres would be swollen significantly. With respect to CH/HA microspheres, HA chains can interact with CH chains via electrostatic force and physical entanglement. Taking into account the low HA/CH ratio and the applied amount of TPP, the resulting CH/HA microspheres could become more compact than CH microspheres and show their SI similar to or slightly smaller than that for CH microspheres. In cases of CH-PLA/HA microspheres, their SI would be mediated by PLA component, CH component as well as TPP. The hydrophobic PCL side chains in CH-PLA chains could turn inward, snag other molecular chains and aggregate inside the microspheres whereas the CH main chains could stretch outward and entangle with HA chains during the preparation of CH-PLA microspheres because a water-based solvent was applied. The entanglements formed among these components would reside inside the microsphere and resist the microsphere swelling, giving rise to their reduced *SI*.

### 3.4. Release Profiles of Microspheres

In principle, multiple factors involving in the structure, morphology and physicochemical property of the drug-loaded microspheres can exert complicated impacts on the release patterns of the loaded drugs [35,36,37]. In this study, the major processing parameters and the compositions for CH-PLA/HA microspheres were already optimized to achieve optional microspheres that meet basic requirements for the delivery of TGF-β1. On this basis, the release profiles of the microspheres were examined to screen out the optimal one.

**Figure 4:**
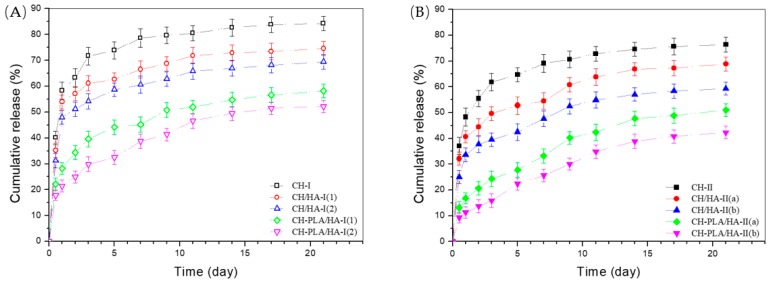
Release profiles of TGF-β1 from microspheres in set-one (**A**) and set-two (**B**) (*n* = 3).

Figure 4 represents the time-dependent TGF-β1 release profiles for different microspheres. CH microspheres in both sets had fast release rates and the cumulative TGF-β1 amount released from these microspheres reached around 70% or even higher in one week. CH/HA microspheres in each set showed reduced release rate within the same sampling time interval when compared to CH microspheres. In Figure 4A, CH/HA-I(2) microspheres showed their release pattern very similar to that for CH/HA-I(1) microspheres without significant difference (*p* > 0.05); but in Figure 4B, the release profile for CH/HA-II(b) microspheres notably differed from that for CH/HA-II(a) microspheres with measurable difference (*p* < 0.05). Based on the data provided in Table 2, it can be drawn that at a low TPP dosage, the effect of HA component on the release behavior of CH/HA microspheres is insignificant but at a high TPP dosage, the HA component could combine with TPP to significantly regulate the release pattern of the CH/HA microspheres. In contrast to these observations, CH-PLA/HA microspheres behaved in different manners. CH-PLA/HA microspheres in both sets showed greatly reduced initial release as compared with CH or CH/HA microspheres in the corresponding set and their TGF-β1 release rate could be conjointly modulated by the HA content and the applied TPP dosage. Among them, CH-PLA/HA-II(b) microspheres could serve as a suitable carrier for the delivery of TGF-β1 since after the first day, these microspheres are able to control the release rate of TGF-β1 in an approximately linear fashion for around 2 weeks.

These results are reasonable if more details for the microspheres are figured out. In principle, the release of drugs or bioactive reagents from polymeric matrices usually involves in swelling, diffusion, swelling followed by diffusion and erosion [10,38]. CH microspheres had high SI because of the loose ionic linkages among CH chains and the chain network is unable to effectively hinder TGF-β1 molecules from rapidly defusing into the surrounding media, leading to their high initial burst and subsequent fast release. TGF-β1 molecules inside CH/HA microspheres face somewhat different situations. At physiological pH, TGF-β1 will be positively charged and can interact electrostatically with negatively charged HA molecules. In addition, HA molecules will also entangle with CH chains due to physical or electrostatic interactions. Therefore, TGF-β1 molecules would be held by both CH and HA and are relatively difficult to be released by diffusion, giving rise to their reduced release rates. In the case of CH-PLA/HA microspheres, the CH-PLA component contains PLA side chains that have high hydrophobicity and low degradation rate and hence, in addition to the mentioned interactions occurred in CH or CH/HA microspheres, TGF-β1 molecules loaded in CH-PLA/HA microspheres will be dragged by PCL side chains and trapped inside the microspheres during the microsphere fabrication, which will cause increased resistance to TGF-β1 molecules and slow them release rate.

In the therapeutic model involving cartilage defects, the applied TGF-β1 dosage needs to be low for functioning as a chemotaxis agent in the early stage and thereafter, to be sufficient for serving as an effective chondrogenic agent until the repair is completed [13,39]. Results in Figure 4 indicate that TGF-β1-loaded CH or CH/HA microspheres are unsuitable for applications in cartilage repair because of their severe initial burst release features and fast release rates. On the other hand, CH-PLA/HA-II(b) microspheres show promising potential for controlling the TGF-β1 release in the desired manner to meet the requirements in cartilage repair.

### 3.5. TGF-β1 Bioactivity

**Figure 5:**
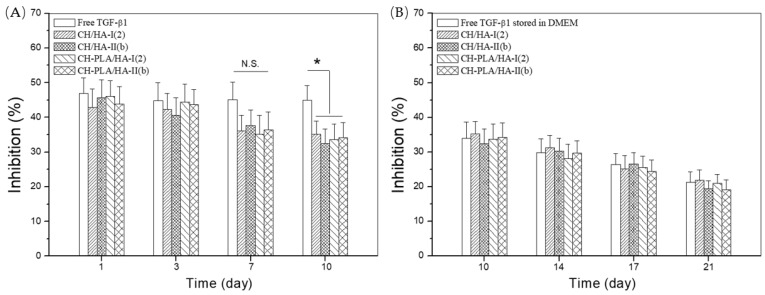
Percent mink lung cell growth inhibition (**A**, free TGF-β1 without storing treatment; and **B**, free TGF-β1 stored in DMEM (dulbecco’s modified eagle medium) at 37 °C for different time intervals changing from 10 to 21 days) of TGF-β1 released from different microspheres during varied periods (*n* = 3; N.S. no significance; * *p* < 0.05).

Four kinds of microspheres were selected from two sets of microsphere samples for the bioactivity assessment of the released TGF-β1 considering that they had higher HA content and were crosslinked with a higher TPP/matrix ratio in comparison to the corresponding ones in each set. Relevant results for cell growth inhibition are graphed in Figure 5. Bar-graphs in Figure 5A indicate that cell inhibition was higher than 35% before day 7 and there were no significant differences in the cell growth inhibition among different sample groups. Nevertheless, it can be seen that the cell growth inhibition detected from microsphere groups at day 10 was shown to be significantly lower than that of free TGF-β1 sample group, meaning that the released TGF-β1 that was collected at day 10 had lower bioactivity when compared with free TGF-β1. 

Significant activity loss of the released TGF-β1 has been detected from some polylactide and poly(dl-lactide-*co*-glycolide) microspheres [40,41]. These polyester microspheres are usually prepared by using organic solvents and their high hydrophobicity and the involved solvents could denature the bioactivity of protein factors [7,10]. In the present study, the used polysaccharides are highly hydrophilic and the microspheres were prepared using an aqueous solvent. Therefore, the employed materials and the microsphere preparation method would not significantly impair the activity of the loaded TGF-β1. In the present case, one possible reason for the activity loss of the released TGF-β1 is that the growth factor lost its functionality by autolysis, as mentioned in some studies [40,42].

In this study, the supernatant collected at day 10 contained a cumulative amount of TGF-β1 that was released from the microspheres during 10 days. TGF-β1 is a type of protein factor and the released TGF-β1 in the supernatant might be autolyzed by way of proteolysis to some extent during this period of time, resulting in its lower activity than free TGF-β1. To clarify whether this explanation is reasonable, the free TGF-β1 was stored in DMEM at 37 °C for the matched periods of time up to 21 days so that it was already exposed to the same environment for the same time interval in comparison to the released TGF-β1 before they both were used for the cell tests. The bioactivity of the released TGF-β1 was further examined for an extended period of time to make additional comparisons and relevant results are presented in Figure 5B. It can be seen that the cell growth inhibition matching with the store-treated free TGF-β1 progressively decreased as time advanced, verifying that the bioactivity of free TGF-β1 would gradually lose if it is exposed to the hydrolysis environment for a period of time longer than a threshold value. It is worth noting from Figure 5B that the released TGF-β1 from different microsphere samples were able to inhibit the cell growth at the same level as compared with the store-treated free TGF-β1 without showing significant differences, indirectly conforming that the activity loss of the released TGF-β1 is not arisen from the employed materials as well as the used microsphere preparation method but possibly from proteolysis. On the basis of results presented in Figure 5, it can be concluded that the presently developed CH-PLA/HA microspheres are reliable for effectively preserving the bioactivity of released TGF-β1.

## 4. Conclusions

Chitosan(CH)-polylactide(PLA) copolymers with PLA side chains at the C-6 sites of CH backbone and good solubility in certain water-based solvents could be used to combine with polyanionic hyaluronic acid (HA) for preparing CH-PLA/HA complexes. Simply blending CH-PLA component with HA component in aqueous phase at formulated ratios would generate irregularly shaped CH-PLA/HA particles or CH-PLA/HA agglomerates and this simple blending method was found to be unpractical for preparing CH-PLA/HA complex microspheres with regular sphericity and controlled sizes. The proposed emulsification preparation method was demonstrated to be successful for constructing TGF-β1-loaded CH-PLA/HA complex microspheres and the resulting microspheres could be endowed with controllable sizes and high encapsulating efficiency. Under optimized preparation conditions, the structures and properties of the CH-PLA/HA microspheres would be synergistically regulated by the PLA component, the applied dose of crosslinker and the incorporated HA amount. The optimal TGF-β1-loaded CH-PLA/HA microspheres had symmetrical size-distribution as well as narrow distribution interval and were able to effectively administrate TGF-β1 release in approximately linear manners for around 2 weeks without showing severe initial burst release features. The released TGF-β1 was detected to be bioactive as compared with the free TGF-β1. Results suggest that these TGF-β1-loaded CH-PLA/HA microspheres had potential in cartilage repair applications due to their controlled TGF-β1 release features and good TGF-β1 bioactivity preservation.

## Figures and Tables

**Table 1:** Parameters of CH-PLA copolymers (*n* = 3).

Copolymer Name	Feed Ratio of LA to PHCH (Molar Ratio)^ (a)^	PLA percentage in CH-PLA (wt %)^ (b)^	Solubility ^(c)^	
			DMSO	Acetic Acid (1.0%)
CH-PLA(1)	2/1	23.6 (±1.51)	−	+
CH-PLA(2)	4/1	38.7 (±1.46)	±	+
CH-PLA(3)	6/1	46.1 (±1.64)	±±	±±
CH-PLA(4)	8/1	52.4 (±1.71)	±±	±±

^(a)^ Ratio of LA to glucosamine units in PHCH (phthaloyl-chitosan); ^(b)^ Contents of C, H and N in the CH-PCLs were measured using an elemental analyzer and the PLA content in CH-PLAs was estimated using the C/N ratio; ^(c)^ “−”, “±”, “±±” and “+” denote that CH-PLAs are insoluble, swelled, partially soluble or highly swelled and soluble, respectively, and DMSO refers to dimethyl sulfoxide.

**Table 2:** Parameters of TGF-β1-loaded microspheres (*n* = 4) ^(†)^.

Microsphere Name	Feed Ratio of HA to CH-PLA (mg/mg)	Feed Ratio of TPP to Matrix (mg/mg)	Average Size (µm)	*EE* (%)	*SI* (%)
CH-I ^ 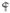 ^	−	2/1	3.73 (±0.41)	29.6 (±3.4)	69.7 (±5.1)
CH/HA-I(1)	0.15/1.0	2/1	3.72 (±0.35)	35.5 (±2.7)	61.5 (±4.6)
CH/HA-I(2)	0.3/1.0	2/1	3.74 (±0.32)	40.7 (±2.9)	59.1 (±3.9)
CH-PLA/HA-I(1)	0.15/1.0	2/1	3.86 (±0.29)	55.2 (±2.8)	46.4 (±3.5)
CH-PLA/HA-I(2)	0.3/1.0	2/1	4.08 (±0.25)	61.4 (±2.5)	44.6 (±3.1)
CH-II ^ 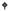 ^	−	4/1	3.16 (±0.27)	37.1 (±3.3)	61.2 (±4.8)
CH/HA-II(a)	0.15/1.0	4/1	3.46 (±0.31)	46.8 (±3.1)	53.4 (±4.3)
CH/HA-II(b)	0.3/1.0	4/1	3.63 (±0.28)	51.6 (±3.6)	48.5 (±3.6)
CH-PLA/HA-II(a)	0.15/1.0	4/1	3.83 (±0.24)	75.3 (±3.2)	32.3 (±3.4)
CH-PLA/HA-II(b)	0.3/1.0	4/1	4.16 (±0.28)	81.9 (±3.8)	30.2 (±3.2)

^(†)^ CH-PLA with a PLA percentage of 38.7 wt % (see Table 1) was used for preparing all kinds of microspheres; ^(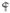,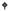)^ Different sample sets: set-one ^(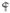)^ and set-two ^(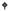)^.
